# Critical Limb Ischaemia in Octogenarians: Treatment Outcomes Compared With Younger Patients

**DOI:** 10.1016/j.ejvsvf.2023.12.003

**Published:** 2024-01-03

**Authors:** Amaia Ormaechevarria, Melina Vega de Céniga, June Blanco, Laura Yáñez, June Fernández, Luis Estallo

**Affiliations:** Department of Angiology and Vascular Surgery, Galdakao-Usansolo University Hospital, Bizkaia, Spain

**Keywords:** Chronic limb threatening ischaemia, Endovascular therapy, Octogenarian, Open surgery, Survival, Limb salvage

## Abstract

**Objective:**

A growing proportion of patients with chronic limb threatening ischaemia (CLTI) are elderly, the most challenging for management decisions. The aim was to study the patient profile and outcome of CLTI in octogenarian patients, comparing them with younger patients.

**Methods:**

Retrospective cohort of consecutive patients hospitalised for CLTI with infrainguinal disease in a Spanish centre (2013–2020). Data on age, comorbidity, anatomical characteristics, and treatment were gathered. Patients were stratified according to age (<80 and ≥80 years). The primary outcomes were overall survival and limb salvage (LS), analysed using Kaplan–Meier and Cox regression.

**Results:**

: A total of 512 patients were enrolled: 305 were <80 years old with mean age 69.7 ± standard deviation (SD) 8.2 years, and 207 were ≥80 years old with mean age 85.3 ± SD 3.6 years. Smoking and diabetes mellitus were more frequent in younger patients (78.0% *vs*. 45.4%, *p* < .001; 68.5% *vs.* 59.5%, *p* = .037 respectively). Older patients had a higher prevalence of heart and kidney disease (70.5% vs. 57.0%, *p* = .002; 39.6% *vs.* 24.3%, *p* < .001, respectively). The arterial disease was femoropopliteal or tibial in 68.9% and 31.1% in patients <80 years and 58.9% and 41.1% in patients ≥80 years (*p* = .021). In younger patients, conservative treatment was indicated in 18.0%, endovascular treatment (ET) in 41.6%, and open or hybrid surgery (OS) in 40.3%; in patients ≥80 years these were 36.9%, 37.4%, and 25.7%, respectively (*p* <. 001). Mean follow up was 23.3 ± SD 17.4 months. One and two year overall survival was 85.4% and 73.0% in younger patients and 64.1% and 51.3% in patients ≥80 years (*p* < .001). LS was 83.7% and 79% at the same times in younger patients and 75.3% and 72.1% in older ones (*p* = .045). In younger patients ET led to worse LS than OS (*p* = .005) but not in older patients (*p* = .29).

**Conclusion:**

Patients ≥80 years with CLTI have higher comorbidity and lower life expectancy and receive conservative treatment more frequently than younger patients. ET and OS are associated with similar survival and LS in these older patients.

## Highlights

• Patients 80 years or older with CLTI receive conservative treatment more frequently than younger patients.
• The results of endovascular and open revascularisation enjoy similar survival and limb salvage rates in this population.
• ET and OS are feasible options in this age group, requiring global assessment and individualised treatment for each patient.

## Introduction

Demographics in Spain show progressive ageing. According to the National Institute of Statistics,^1^^,^^2^ the octogenarian population in Spain is expected to double by 2050, reaching 11.6% of the general population.

The prevalence of peripheral arterial disease (PAD) increases progressively with age. In the United States, it is estimated to affect 3–10% of the population over 50 years of age,^3^^,^^4^ 20–25% in the population over 70 years, and around 20% in those over 90 years, being asymptomatic in 80% of the latter.^5^^,^^6^ In Spain, the prevalence of PAD is estimated at 15–20% in people over 70 years old.^7^^,^^8^ Chronic limb threatening ischaemia (CLTI) represents the most advanced and severe phase of the disease with rest pain, ulcers, or distal gangrene and affects about 11% of patients with PAD. Without revascularisation, the incidence of amputation is approximately 25% a year after diagnosis.^9^^,^^10^

Thus, an increasingly elderly population with greater pathology and prevalence of concomitant heart disease, and renal or respiratory disease is being treated,^3^ which requires personalised management adjusted to their comorbidities, surgical risk, life expectancy, and quality of life. For this, it is important to know the characteristics of the pathology and the results of the different treatment strategies specifically in this elderly population group.

The characteristics and outcomes of CLTI in octogenarian and nonagenarian patients is analysed and compared with younger patients.

## Materials and methods

A retrospective study of a prospective cohort of consecutive patients hospitalised for CLTI with infrainguinal disease from 2013 to 2020 was performed.

The following data were gathered: age, cardiovascular risk factors (smoking, hypertension, hypercholesterolemia, diabetes mellitus [DM]), comorbidity (heart disease, chronic obstructive pulmonary disease, chronic kidney disease [CKD], cerebrovascular disease, concomitant abdominal aortic aneurysm, cancer, previous revascularisation procedures, characteristics of the PAD [stage, location]), and the treatment the patient received.

Smoking was defined as both former and active. Hypertension was defined as blood pressure >140/90 mmHg in baseline conditions or if the patient was taking hypotensive medication, and hypercholesterolaemia if the patient had basal total cholesterol >200 mg/dL and or low density lipoprotein >100 mg/dL or if the patient was being treated with lipid lowering medication and or a specific diet. DM was defined by a fasting glucose ≥126 mg/dL, glycated haemoglobin >6.5%, or the patient was taking oral antidiabetics and or insulin.

Regarding comorbidities, heart disease included history of angina and or acute myocardial infarction, revascularised (endovascular or open surgery) or not, valve disease, heart failure, dysrhythmia, and non-ischaemic cardiomyopathy (idiopathic or alcoholic). Patients with chronic obstructive pulmonary disease had been diagnosed by a respiratory physician and or were under treatment with inhalers. CKD was defined by serum creatinine ≥1.5 mg/dL and or glomerular filtration rate <60 mL/min/1.73 m^2^. Cerebrovascular disease included a history of stroke, transient ischaemic attack and or moderate to severe carotid stenosis. Concomitant abdominal aortic aneurysm was diagnosed when the maximum infrarenal aortic diameter was ≥30 mm. For cancer history, both active and treated neoplasms in remission were taken into account for any aetiology.

The stages of CLTI were Fontaine III, rest pain, or IV, or trophic lesions. The anatomical location of disease was defined as femoropopliteal when the patient presented a normal femoral pulse with absence of popliteal and distal pulses, and significant disease was confirmed in the superficial femoral artery and or the popliteal artery on imaging tests (ultrasound, magnetic resonance imaging, and or computed tomography angiography). Tibial disease was defined as the absence of significant superficial femoral artery or popliteal disease, and disease confirmed and confined to the tibial vessels, verified with ultrasound mapping and magnetic resonance imaging or computed tomography angiography.

Therapeutic strategies were conservative treatment or revascularisation by endovascular therapy (ET), open, or hybrid surgery (OS). Conservative treatment was defined as no revascularisation, and included best medical treatment (antiplatelet or anticoagulant, statin), local wound care, analgesia, debridement, and direct minor (digital, transmetatarsal, Lisfranc) or major (below or above knee) amputation. It was usually selected in non-ambulatory patients, those with severe comorbidity and short life expectancy, patients with no revascularisation options due to the extent of the disease with no run off, patients with limited tissue loss or very distal arterial disease and perfusion considered sufficient or borderline.

Patients were followed until death or 31 December 2021. The outcome variables were overall survival and limb salvage (as defined by no major amputation). End points of major amputation and death were ascertained through hospital records and official census data. The patients were stratified into two groups according to age (<80 years and ≥80 years). Statistical analysis was performed using the chi square and Fisher's exact test for categorical data, Student's t test for continuous data, Kaplan–Meier to estimate survival, and the log rank test and univariable Cox regression to assess differences in survival or limb salvage comparing both age groups, overall and according to the selected treatment, and comparing treatment modalities within each age group. A *p* value <.05 was considered statistically significant.

## Results

Five hundred and twelve patients were included: 305 were <80 years and 207 were ≥80 years. Patients <80 years included 224 men (73.4%) and 81 women (26.6%), with a mean age of 69.7 ± standard deviation (SD) 8.2 (range 36–79.8) years. Patients ≥80 years included 131 men (63.3%) and 76 women (36.7%), with a mean age of 85.3 ± SD 3.6 (range 80–97) years.

The clinical characteristics of the patients are detailed in Table 1. Smoking and DM were more frequent in the younger population with prevalence of 78.0% and 68.5% respectively, compared with 45.4% and 59.5% in the older population (*p* < .05). However, hypertension was significantly more prevalent in the octogenarian population (94.7% *vs.* 85.6%, *p* < .001). Octogenarian and nonagenarian patients had a higher prevalence of heart disease (70.5% *vs.* 57.0%; *p* = .002), CKD (39.6% *vs.* 24.3%; *p* < .001), and cerebrovascular disease (24.6% *vs.* 14.8%; *p* = .005). Younger patients had received previous limb revascularisation (in the same limb or the contralateral limb) more frequently than octogenarians (33.1% *vs.* 21.7%; *p* = .005). Older patients presented with trophic lesions more frequently than the younger patients (90.3% and 81.0%; *p* = .004). The most affected sector was femoropopliteal in both age groups, with octogenarians having distal disease more frequently than the younger population (*p* = .021).

**Table 1:** Clinical characteristics of the total cohort and both age groups (<80 years *vs.* ≥ 80 years), including cardiovascular risk factors, comorbidity, previous limb revascularisation, stage, and anatomical location of the peripheral arterial disease (PAD), and selected treatment.

Characteristic	Total *n* (%)	Patients <80 years *n* (%)	Patients ≥80 years *n* (%)	*p* value
*Cardiovascular risk factors*				
Smoking	331 (64.6)	237 (78.0)	94 (45.4)	<.001
Hypertension	457 (89.2)	261 (85.6)	196 (94.7)	<.001
Dyslipidaemia	391 (76.4)	234 (76.7)	157 (75.8)	.81
DM	331 (64.6)	209 (68.5)	122 (59.5)	.037
*Comorbidity*				
Heart disease	320 (62.5)	174 (57.0)	146 (70.5)	.002
Coronary artery disease	156 (30.5)	101 (33.2)	55 (26.6)	.11
COPD	98 (19.1)	52 (17.0)	46 (22.2)	0.14
CKD	156 (30.5)	74 (24.3)	82 (39.6)	<.001
Cerebrovascular disease	96 (18.8)	45 (14.8)	51 (24.6)	.005
AAA	15 (2.9)	9 (3.0)	6 (2.9)	.97
Cancer	78 (15.2)	44 (14.4)	34 (16.4)	.54
*PAD*				
Previous limb revascularisation	146 (25.5)	101 (33.1)	45 (21.7)	.005
Fontaine stage				.004
III	78 (15.2)	58 (19.0)	20 (9.7)	
IV	434 (84.8)	247 (81.0)	187 (90.3)	
*Disease location*				.021
Femoropopliteal	332 (64.8)	210 (68,9)	122 (58.9)	
Tibial	180 (35.1)	95 (31.1)	85 (41.1)	
*Selected treatment*				<.001
Conservative	131 (25.6)	55 (18.0)	76 (36.9)	
Endovascular	204 (39.8)	127 (41.6)	77 (37.4)	
Open or hybrid	176 (34.7)	123 (40.3)	53 (25.7)	

Data are presented as *n* (%). AAA = abdominal aortic aneurysm; CKD = chronic kidney disease; COPD = chronic pulmonary obstructive disease; CVRF = cardiovascular risk factors; DM = diabetes mellitus; PAD = peripheral arterial disease.

In patients <80 years, conservative treatment was selected in 18.0%, endovascular treatment (ET) in 41.6%, and open or hybrid revascularisation (OS) in 40.3%. In the population ≥80 years, corresponding frequencies were 36.9%, 37.4%, and 25.7%, respectively (*p* < .001) (Table 1). The revascularisation techniques in each group are detailed in Table 2.

**Table 2:** Revascularisation techniques in the cohort in patients <80 years and ≥80 years.

Treatment	Revascularisation techniques	*n (%*)
*Patients < 80 years old*		
Endovascular treatment	PTA	89 (29.2)
	PTA + stent	37 (12.1)
Hybrid or open surgery	Thrombectomy	2 (0.7)
	ATK femoropopliteal bypass	19 (6.2)
	BTK femoropopliteal vein bypass	27 (8.9)
	BTK femoropopliteal prosthetic bypass	3 (1.0)
	Femorodistal vein bypass	26 (8.5)
	Femorodistal prosthetic bypass	2 (0.7)
	Popliteodistal bypass	6 (2.0)
	Femoral endarterectomy	38 (12.5)
*Patients ≥ 80 years*		
Endovascular treatment	PTA	63 (30.4)
	PTA + stent	13 (6.5)
Hybrid or open surgery	Thrombectomy	1 (0.5)
	ATK femoropopliteal bypass	6 (2.9)
	BTK femoropopliteal vein bypass	12 (5.8)
	BTK femoropopliteal prosthetic bypass	3 (1.4)
	Femorodistal vein bypass	9 (4.5)
	Popliteodistal bypass	4 (1.9)
	Femoral endarterectomy	15 (7.2)
	Popliteal endarterectomy	1 (0.5)

ATK = above knee; BTK = below knee; PTA = percutaneous transluminal angioplasty.

**Figure 1:**
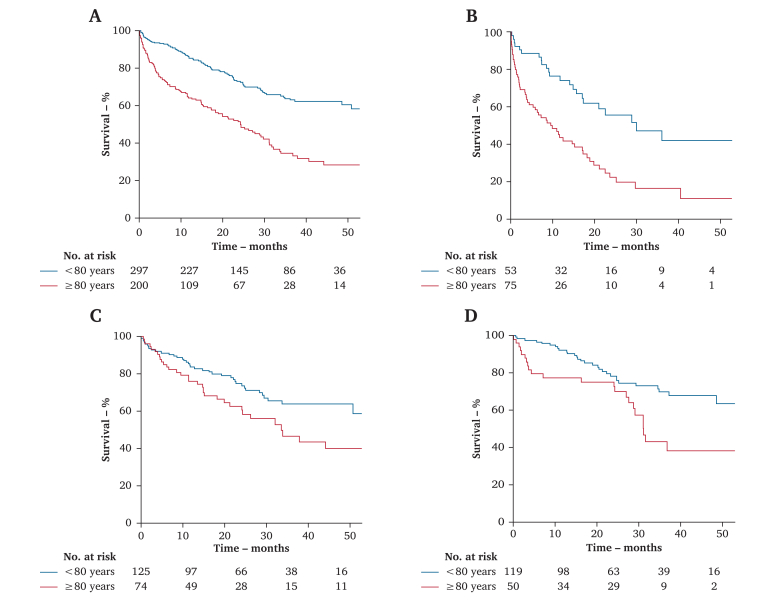
Cumulative Kaplan-Meier estimate of overall survival in patients < 80 years (blue line) and ≥ 80 years (red line). (A) Overall cohort, *p* < .001 (B) Non-revascularised patients (*p* < .001). (C) Patients revascularised by ET (*p* = .037). (D) Patients revascularised by OS (*p* < .001). Tables: patients (n) entering each interval: baseline, 1^st^, 2^nd^, 3^rd^ and 4^th^ year.

Mean follow up was 23.3 ± SD 17.4 (range 0.2–94.3) months. The number of patients lost to follow up was 68 (13.3%). The early (30 day) mortality rate was 4.0% and 4.1% for ET in patients <80 and ≥80 years, respectively (*p* = .99), 1.7% and 4.0% for OS patients (*p* = .37), and 5.7% and 17.3% for conservative treatment (*p* = .049). Overall, one and two year survival was 85.4% and 73.0% in patients <80 years and 64.1% and 51.3% in patients ≥80 years, respectively (*p* < .001) (Fig. 1A). There was better overall survival in younger patients regardless of the selected treatment (Fig. 1B–D).

**Figure 2:**
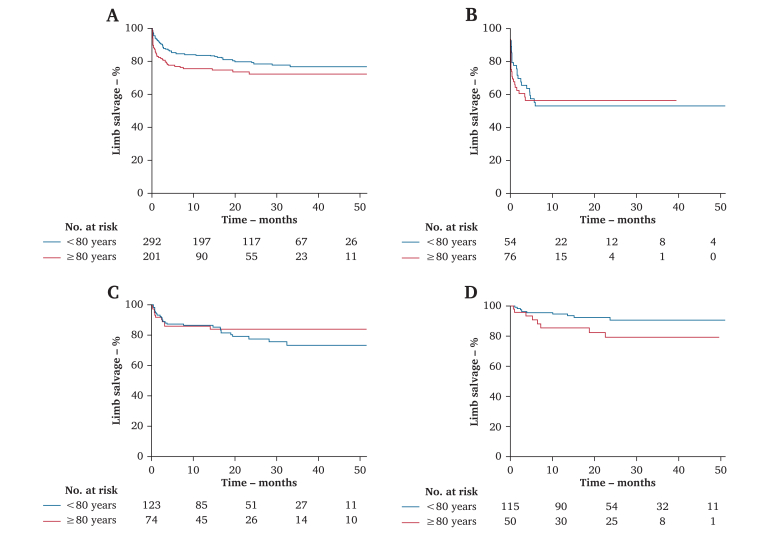
Cumulative Kaplan-Meier estimate of limb salvage in patients < 80 years (blue line) and ≥ 80 years (red line). (A) Overall cohort. (B) Non-revascularised patients (*p* = .71). (C) Patients revascularised by ET (*p* = .47). (D) Patients revascularised by OS (*p* = .082). Tables: patients (n) entering each interval: baseline, 1^st^, 2^nd^, 3^rd^ and 4^th^ year.

Limb salvage was 83.7% and 79.0% after one and two years in patients <80 years and 75.3% and 72.1% in patients ≥80 years (*p* = .045). The maximum risk of limb loss lay in the first six months for both groups, with few events thereafter (Fig. 2A). There was no significant difference in LS in either group treated with conservative management, ET, or OS (Fig. 2B–D).

One hundred and twelve patients (21.9%) required re-intervention for further or new revascularisation, 80 (26.2%) among the younger patients and 32 (15.5%) among the older ones (*p* = .013).

**Figure 3:**
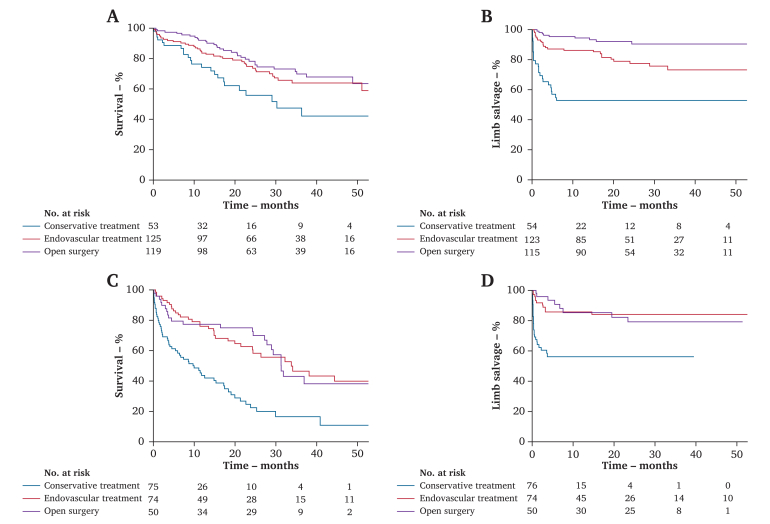
Cumulative Kaplan-Meier estimates of overall survival and limb salvage. (A) Overall survival in patients < 80 years stratified by the selected treatment: conservative (blue line), endovascular (red line), open surgery (purple line). (B) Limb salvage in patients < 80 years stratified by the selected treatment. (C) Overall survival in patients ≥ 80 years stratified by the selected treatment. (D) Limb salvage in patients ≥ 80 years stratified by the selected treatment. Tables: patients (n) entering each interval: baseline, 1^st^, 2^nd^, 3^rd^ and 4^th^ year.

Additional analyses were made according to the type of treatment within each age group. In the younger patients, overall survival was significantly worse in patients who were treated conservatively (*p* = .014), with no difference between those revascularised by ET or OS (*p* = .29) (Fig. 3A). Limb salvage (LS) was worst in patients managed conservatively, and ET led to worse LS than OS (*p* = .005) (Fig. 3B). In the older patients, overall survival was worse in patients managed conservatively (*p* < .001), with no differences in patients revascularised by an endovascular or open procedure (*p* = .99) (Fig. 3C). LS was again worse in patients treated conservatively (*p* < .001), and similar in those treated with ET or OS (*p* = .29) (Fig. 3D).

## Discussion

Two hundred and seven octogenarian and nonagenarian patients with CLTI were studied. They had a lower prevalence of smoking and DM but higher cardiac and renal comorbidity than younger patients. These elderly patients were managed conservatively in over a third of the cases, and the most frequent revascularisation strategy was endovascular. Overall survival was 51.3% after two years, with a 72.1% limb salvage rate. ET and OS obtained similar results in this population.

The octogenarian and nonagenarian patient profiles are slightly different from younger patients, with a lower prevalence of common cardiovascular risk factors, such as smoking and DM, but higher comorbidity of other organs, mainly cardiac and kidney. CLTI also seems to appear later, with a lower frequency of revascularisation procedures, and more common distal territory disease.

Because of the increased comorbidity and therefore surgical risk, and more distal arterial disease, the octogenarian population received conservative management more frequently than younger patients^11^^,^^12^ In the patients <80 years, revascularisation was indicated in 81.9% of the cases, while in only 63.1% of older patients. Factors that influenced this decision included ultradistal disease, short life expectancy, severe comorbidity, small lesions without associated pain, and a multidisciplinary approach to the mechanical and local component of the lesions. The lower life expectancy is reflected in the data, 48.7% of the population ≥80 years died within two years of follow up, reaching almost to two thirds in the third year.

The comorbidity of the elderly patients increases the surgical risk. However, the early mortality rate of 4% was obtained for both ET and OS, not significantly higher than the younger patients. Thorough clinical assessment and patient selection are crucial to ensure patient safety and improve results.

The risk of major amputation is highest in the first six months of follow up, due to the extent of the lesions, uncontrollable pain, or failure of the revascularisation. After the first year, the risk of limb loss is low in both age groups and for all treatment modalities. In younger patients, OS seems to offer better limb salvage results without increasing the mortality rate, so it seems that an ET strategy should not be considered as first option systematically. In elderly patients, both techniques seem to offer similar results and both are options to be considered; the best choice will be individualised, based on clinical and anatomical characteristics, the availability of resources and expertise.

The results seem to be in line with the recently published data of the Best Endovascular *vs.* Best Surgical Therapy in patients with CLTI (BEST CLI) trial.^10^ This international multicentre trial recruited 1830 patients with CLTI and infrainguinal disease, in two different cohorts based on the availability of a valid venous segment as a conduit. Both cohorts were randomised to OS or ET. Cohort 1 (patients with a useable venous segment) had similar mortality rates in the OS and ET groups, and a lower rate of major amputation and limb re-intervention in patients who received OS (hazard ratio [HR] 0.73, 95% confidence interval [CI] 0.54–0.98; and HR 0.35, 95% CI 0.27–0.47, respectively). In cohort 2 (patients lacking a suitable venous segment for bypass), no differences were found in terms of survival, rates of re-intervention, or major amputation between groups, although the patients revascularised with endovascular techniques suffered earlier re-interventions. The subgroup of patients ≥80 years in BEST CLI obtained similar results for OS and ET. The results are consistent with this clinical trial, with better limb salvage for open techniques in younger patients and similar rates for OS and ET in older patients.

In contrast, some studies^13^^,^^14^ have reported higher mortality rates in patients revascularised to OS due to the increased risk of cardiovascular events. In the BASIL trial^15^ similar one and two year overall survival and limb salvage rates were described for ET and OS, with higher costs due to the longer hospital stay in the latter. The data improved in favour of the OS group after two years of follow up. In the recent BASIL 2^16^ trial, in which 345 patients with infrapopliteal disease and life expectancy greater than six months were randomised to ET or OS, ET obtained better amputation free survival (AFS) driven entirely by an excess long term mortality rate in the surgical group. No differences were found between the two groups in terms of comorbidity, 30 day mortality, or acute limb events. However, they did report a higher rate of re-interventions during follow up in the endovascular group.

In the CRITISCH German multicentre Registry, the one year AFS rates for ET, bypass grafting, femoral patch plasty, and conservative treatment were 75%, 72%, 73%, and 72% respectively, with CKD identified as a risk factor for all techniques, and prosthetic conduit and previous vascular intervention as independent risk factors for diminished AFS after bypass surgery.^17^ A study from that same registry focusing on ET in octogenarians compared with younger patients revealed comparable in hospital mortality (2% and 1% respectively) and one year AFS rates (77% and 75% respectively), with better one year limb salvage (95% and 90%, respectively, *p* = .010) and freedom from major adverse limb event (72% and 62% respectively, *p* = .016) rates in the octogenarian group.^18^

This study is limited by the observational nature of the cohort, with inherent bias in treatment selection. Age, life expectancy, comorbidity, surgical risk, the availability of a saphenous vein conduit, and the extent of the disease, both clinical and anatomical, have all influenced the choice of treatment in each patient. Randomised clinical trials provide the greatest quality of evidence but are limited by strict inclusion criteria. The BEST CLI and BASIL-2 trials randomised patients considered to have equipoise for ET and OS, excluding a large range of patients unsuitable for one or the other or both treatments. Registries, in contrast, provide real world data with consecutive inclusion of patients with more heterogeneous clinical and anatomical characteristics. The cohort included a large group of consecutive patients with all possible management strategies, including conservative treatment and primary amputation, and focused on robust outcomes of survival and limb salvage, thus adding to the pool of knowledge from which to drive clinical decisions.

## Conclusion

Patients 80 years or older with CLTI have higher comorbidity and lower life expectancy and receive conservative treatment more frequently than younger patients. The results of endovascular or open revascularisation achieve similar survival and limb salvage rates, thus both are valid and feasible options in this age group. The octogenarian population with CLTI requires holistic assessment and individualised treatment.

## Conflict of interest

None.

## Funding

None.
